# Mark E. Josephson (1943–2017)

**DOI:** 10.1007/s12471-017-0968-1

**Published:** 2017-02-28

**Authors:** R. N. W. Hauer

**Affiliations:** Netherlands Heart Institute, University Medical Center Utrecht, Utrecht, The Netherlands

Dr. Mark Josephson was an innovative giant in clinical electrophysiology. On 11 January 2017 he passed away after a long illness.

**Figure 1:**
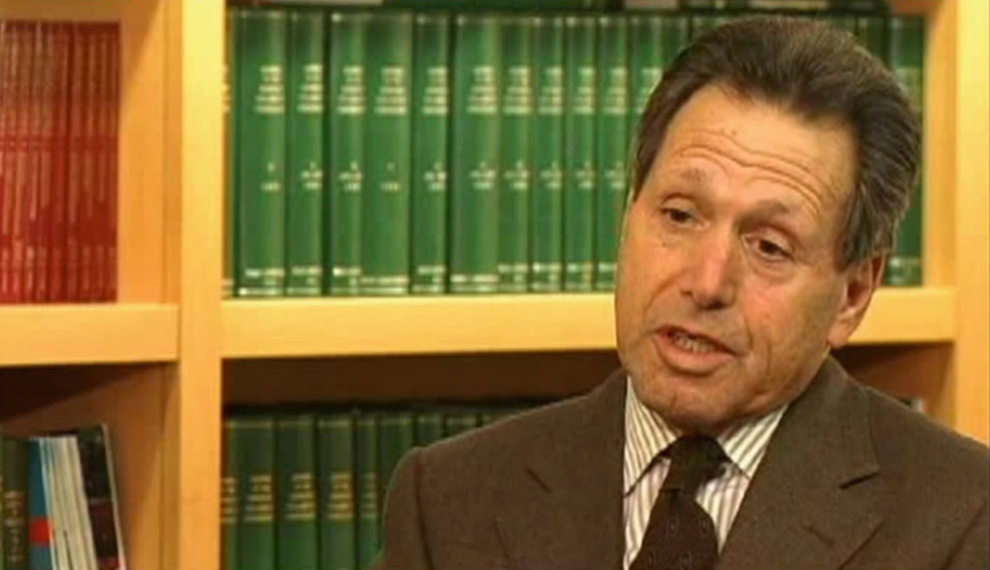
Beth Israel Deaconess Medical Center

Josephson started his scientific carrier in the early 1970s at the electrophysiological laboratory of Dr. Anthony Damato on Staten Island. Later he moved to Philadelphia to start his own laboratory. In 1978 and 1979 he published a series of four famous key papers in *Circulation* on mechanisms and endocardial catheter mapping of sustained ventricular arrhythmias. This paved the way for mapping-directed surgical treatment, and at a later stage for catheter ablation. After awareness of the limited value of pharmacological therapy for ventricular arrhythmias, these invasive therapeutic strategies soon appeared as promising new avenues. As a young inexperienced cardiologist I had the opportunity to visit his lab in 1980, and fascinated by his thoughts and work decided to devote the focus of my thesis on ventricular tachycardia mapping and ablation. Josephson’s last position for many years was professor of Harvard University at the Beth Israel Deaconess Medical Center in Boston.

Josephson is (co)author of more than 500 peer-reviewed publications, mostly in top-ranking journals. His meticulous analysis of intra-cardiac electrograms contributed tremendously to improvement of ECG interpretation, and thus patient care. He was not only an excellent scientist, but above all an outstanding teacher. His systematic textbook *Clinical Cardiac Electrophysiology: Techniques and Interpretation*, a voluminous single-author work with four editions, was and still is the major reference work on many different aspects of clinical electrophysiology for numerous fellows, as well as experienced electrophysiologists worldwide. Together with Hein Wellens, high-level 5‑day training courses on ECG and intra-cardiac electrogram interpretation were organised several times a year since 1981, in Europe as well as in the United States.

We will miss his enthusiasm and continuous support. Cardiology has lost a great man.

